# The Rhode non-linearity and its impact on cochlear mechanics

**DOI:** 10.1121/10.0017073

**Published:** 2023-02-02

**Authors:** Sunil Puria, John J. Guinan

**Affiliations:** 1 Mass Eye and Ear, Boston, Massachusetts 02114, USA; 2 Harvard Medical School, Boston, Massachusetts 02115, USA

## Abstract

The *Reflections* series takes a look back on historical articles from *The Journal of the Acoustical Society of America* that have had a significant impact on the science and practice of acoustics.

## ARTICLE BACKGROUND

Before Rhode's classic paper was published 50 years ago,^1^ the cochlea was thought to be a linear, time-invariant mechanical system that drove a nonlinear neural system. The Nobel-Prize-winning work of von Bekesy^2^ showed that a tone produced a traveling wave on the basilar membrane (BM) that peaked at more apical locations for lower frequencies. However, von Bekesy's measurements were on cadaver ears. To enable measurements on live ears, Johnstone and Boyle developed a new method for measuring the small cochlear motions, the “Mossbauer method” that used the Doppler shift of nuclear radiation from a source put on the BM.^3^ They found V-shaped tuning curves that were slightly narrower than von Bekesy's.^3^ With the view that cochlear mechanics was linear, this was interpreted as showing that von Bekesy's results also applied to living ears.^4^ At this time, however, there were other results that were difficult to interpret with the view that cochlear mechanics was linear.^5,6^

## ARTICLE OVERVIEW

Rhode's results were the first measurements that showed that BM motion grew nonlinearly at frequencies near the best frequency. A key thing that Rhode did was to measure BM motion at multiple levels (if the cochlea was linear, a measurement at one level was all that was needed). The iconic figure from Rhode's work is his Fig. 6 (shown here, Fig. 1) which graphs responses from three levels as gains, i.e., BM displacement divided by malleus displacement. At low frequencies the gain was the same across level (as would be true in a linear system) but near the best frequency the gain became less when level was increased. Rhode's Fig. 7 showed that at lower levels, near-BF BM responses became linear, so the nonlinearity was level dependent. While his landmark measurements of BM responses at different stimulus levels were made on several animals, the paper only showed results in a single animal.

**Figure:**
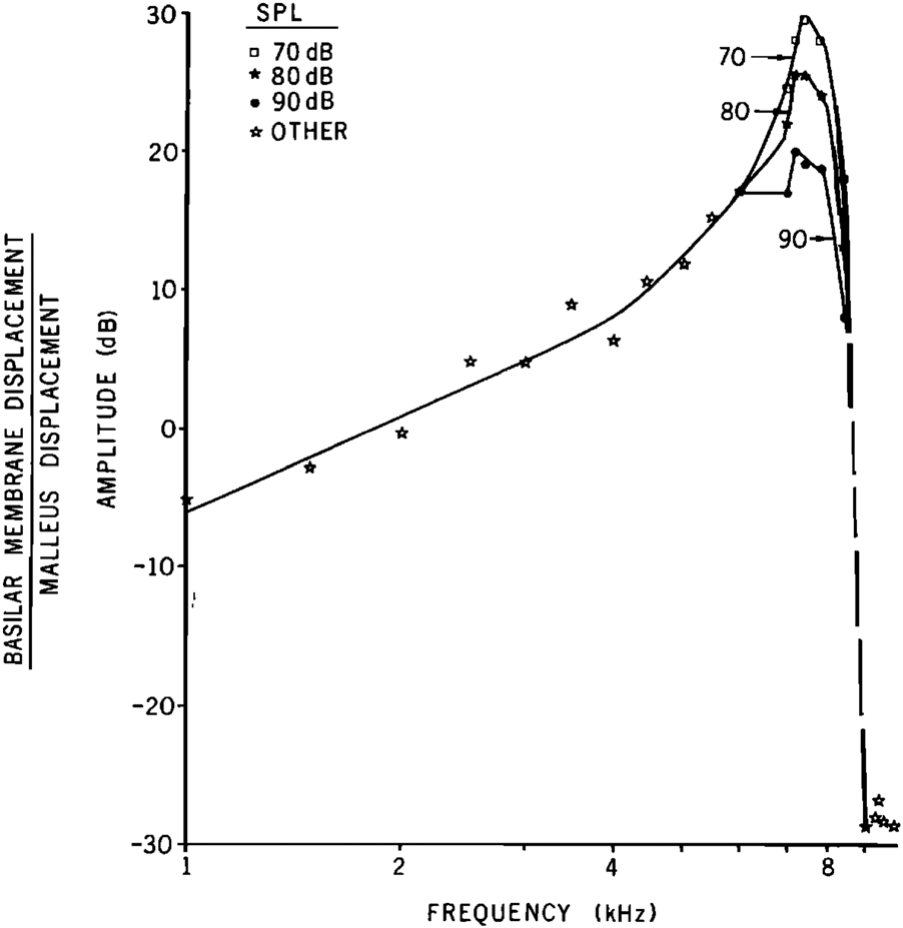
Amplitude of the ratio in dB of the displacements of the basilar membrane and malleus for three SPLs, 70, 80, and 90 dB (re 0.0002 dyn/cm2). Reprinted with permission, J. Acoust. Soc. Am. **49**, 1218–1231 (1971). Copyright 1971 Acoustical Society of America (Ref. 1).

## IMPACT OF THE ARTICLE

Rhode's discovery, that the mechanical tuning of the organ of Corti is non-linear, has stood the test of time and has been reproduced many times. After Rhode's seminal work, two other discoveries have also shaped the modern view of cochlear mechanics: (1) Otoacoustic emissions:^7^ Sounds of cochlear origin transmitted backward through the middle ear and recorded in the ear canal and (2) Outer hair cell (OHC) motility:^8^ that OHCs through their piezoelectric-like properties could exert forces at frequencies of tens of kHz. With the advent of optical coherence tomography, which has shown that the organ of Corti above the BM moves even more than the BM, the field of cochlear mechanics is going through another revolution.^9–12^ Nonetheless, Rhode's work marks the onset of the modern era of cochlear mechanics.

Rhode's physiological measurements had an impact on many areas including psychoacoustics. In the early days at Bell Labs, Harvey Fletcher mapped out the power law relation between intensity and loudness.^13^ It was already known that the auditory nerve had a dynamic range (about 60 dB) that was significantly less than that of the much larger dynamic range of ear canal sound pressures (about 120 dB). Rhode's measurements showed that one site of this dynamic range compression is BM non-linearity. Significant activity followed and we now know that the site of this non-linearity are the outer hair cells that are physiologically vulnerable. Noise and chemical insults to these hair cells renders the BM linear as observed by Bekesy.
